# Antimicrobial and Herbal Drug Resistance in Enteric Bacteria Isolated from Faecal Droppings of Common House Lizard/Gecko (*Hemidactylus frenatus*)

**DOI:** 10.1155/2013/340848

**Published:** 2013-09-24

**Authors:** Bhoj R. Singh, Vidya Singh, N. Ebibeni, Raj K. Singh

**Affiliations:** ^1^ICAR Research Complex for NEH Region, Jharnapani, Nagaland 797 106, India; ^2^I/C Epidemiology Section, Indian Veterinary Research Institute, Izatnagar 243122, India; ^3^NRC on Mithun, Jharnapani, Nagaland 797 106, India

## Abstract

From 194 faecal dropping samples of common house geckos collected from offices (60), houses (88), integrated farm units (IFS,18) and hostels, guest houses, and dining rooms of different canteen/mess (HGM, 28), 326 bacterial isolates of enteric bacteria belonging to 17 genera and 34 species were detected. *Escherichia coli *were the most frequently (39) isolated followed by *Citrobacter freundii *(33), *Klebsiella pneumonia *(27), *Salmonella indica *(12), *Enterobacter gergoviae *(12), and *Ent. agglomerans *(11). Other important bacteria isolated from gecko droppings were *Listonella damsela *(2), *Raoultella terrigena *(3), *S. salamae *(2), *S. houtenae *(3), *Edwardsiella tarda *(4), *Edwardsiella hoshinae *(1), and *Klebsiella oxytoca *(2). Of the 223 isolates tested for antimicrobial drug sensitivity, 27 (12.1%) had multiple drug resistance (MDR). None of the salmonellae or edwardsiellae had MDR however, MDR strains were significantly more common among *Escherichia *spp. (*P* = 1.9 × 10^−5^) and isolates from IFS units (*P* = 3.58 × 10^−23^). The most effective herbal drug, *Ageratum conyzoides *extract, inhibited growth of only 27.8% of strains tested followed by ethanolic extract of *Zanthoxylum rhetsa *(13.9%), eucalyptus oil (5.4%), patchouli oil (5.4%), lemongrass oil (3.6%), and sandalwood oil (3.1%), and *Artemisia vulgaris* essential oil (3.1%).

## 1. Introduction

In most parts of the world house wall lizards are common. In the Jharnapani area, common house gecko (*Hemidactylus frenatus*) is found everywhere in houses, in animal sheds, in offices, and so forth. Geckos are often reported as carriers of many zoonotic enteropathogens including nontyphoidal salmonellae [1–5], *Citrobacter freundii, C. Intermedius, Erwinia herbicola, Enterobacter cloacae* [2, 5], *Shigella sonnei, Edwardsiella tarda, Enterobacter* species, *Serratia marcescens, Proteus* spp., *Klebsiella pneumonia*, and *Escherichia coli* [5]. Researchers have suspected that lizards have a role as reservoirs in spread and emergence of drug resistant bacteria [2, 4, 5]. However, most of the studies on enteropathogens of public health significance in lizards have been conducted through collecting lizards, euthanizing them, and then collecting their intestinal contents, determining the presence of bacteria.

Enteric diseases often spread through contamination of environment with the faeces of patients loaded with the pathogens. Insects often try to scavenge on patients' excreta, and those insects that are eaten by house geckos may be sources of different pathogens in lizard intestine and may be detected in lizards' intestine [2, 5]. The question we asked was what bacteria of public health concern are present in excreta of lizards which may be source of reinfection to human and animals. Though earlier studies are impressive, it is known that bacteria detected in intestines may not always be present in the excreta probably due to several competitive factors in posterior part of gastrointestinal tract. Therefore, we attempted to determine enteric bacteria in droppings of geckos instead of collecting them from intestines. Another query in the study was to determine the effect of the environment on bacteria present in gecko excreta. To this end, samples were collected from four different environments. Further, we determined the antimicrobial drug sensitivity of bacterial isolates from faecal dropping of geckos.

## 2. Materials and Methods

### 2.1. Samples

Faecal dropping of common house lizards (geckos) were collected taking care that it does not come in human or animal contact before, during or after collection of samples. To collect samples sterile masks and gloves were worn and gloves were changed after every sample. One sample constituted all the droppings collected in one enclosure/animal house, or a family home. Up to 10 droppings which had not fallen on ground, that is, still sticking on wall or roof of the room and apparently fresh, were collected in sterile 50 mL tube with the help of sterile forceps. In total 194 samples were collected in and around Jharnapani, Dimapur, Nagaland, India, in the months from March to June. Samples were collected from 60 offices (banks, administrative offices of district, the National Research Centre on Mithun, and the ICAR Research Complex, Jharnapani); 88 households in and around Jharnapani village of Dimapur district, residential quarters of the National Research Centre on Mithun and ICAR Research Complex, Jharnapani; 18 units of integrated farms (IFS) rearing pigs (6), rabbits (4), cows (6), ducks (1) and vegetable farming (1); 24 hostel and guest house rooms at the National Research Centre on Mithun and the ICAR Research Complex, Jharnapani; and four dining rooms (one each in a hostel, guest house, the ICAR canteen, and a the CISF mess at Jharnapani) (HGM).

### 2.2. Processing of Samples and Identification of Bacteria

Samples were brought to the laboratory within an hour of collection and processed for isolation of different bacteria. To each tube 20 mL of sterile buffered peptone (BPW, Hi-Media, Mumbai) water was added. Tubes were incubated at 37°C for 2 h, then swirled with a vortex mixer to mix the contents of tube uniformly, and again incubated for 6–8 h at 37°C. Growth from the tubes was streaked on to MacConkey agar (MA, Hi-Media) and Hektoen enteric agar (HEA, Hi-Media), and plates were incubated for 24–36 h at 37°C. An example of each visibly different type of isolated colonies was picked and restreaked on brain heart infusion agar (BHIA, Hi-Media) for final isolation of bacteria. From BHIA, a single colony from each isolate was characterised using morphological, cultural, staining, and growth parameters as well as using Hi-Assorted biochemical test kit (Hi-Media) and Hi25 *Enterobacteriaceae* identification kit (Hi-Media) as described by the manufacturer.

### 2.3. Antimicrobial Sensitivity Assay

Selected isolates of different bacteria were tested for antimicrobial sensitivity using disc diffusion method [6] on the Muller Hinton agar (MHA, Hi-Media) plates against antimicrobial discs (Hi-Media) of ampicillin (10 *µ*g), cefotaxime (30 *µ*g), chloramphenicol (30 *µ*g), ciprofloxacin (30 *µ*g), cotrimoxazole (25 *µ*g), gentamicin (10 *µ*g), nitrofurantoin (300 *µ*g), and tetracycline (30 *µ*g), and the results were interpreted as per guidelines of CLSI [6]. Isolates resistant to three or more drugs were classified as multidrug-resistant (MDR) type.

The disc diffusion method, as described earlier [7, 8], was followed for determining sensitivity of bacterial strains against herbal antimicrobials including *Ageratum conyzoides* ethanolic extract (AC), *Artemisia vulgaris* essential oil (AV), eucalyptus gum (EG), lemongrass oil (LGO), patchouli essential oil (PO), sandal wood oil (SWO), and *Zanthoxylum rhetsa* ethanolic extract (EO). For testing herbal drug sensitivity discs were prepared to contain 500 *µ*g of herbal preparations using sterile blank 6 mm discs (Hi-Media) and herbal extracts/oils kindly provided by Naga Fragrance Pvt. Ltd. Dimapur, Nagaland. 

### 2.4. Statistical Analysis

All bacteria isolation and sensitivity assay data was entered in MS Excel work sheet and analysed to evaluate the effect of different sources of geckos on the type of bacterial strains isolated from faecal droppings and their sensitivity to antimicrobial drugs using the two-tailed Chi-square test.

## 3. Results

### 3.1. Bacterial Isolates from Gecko Droppings

From 194 faecal dropping samples of common house geckos collected from different places, 326 bacterial isolates were identified as belonging to 17 genera and 34 species (Table 1). *Escherichia coli *was isolated from the highest number of samples (39) followed by *Citrobacter freundii* (33), *Klebsiella pneumonia* (27), *Salmonella indica* (12), *Enterobacter gregoviae* (12), and *Ent. agglomerans* (11). Other bacteria belonging to 28 species were detected in only a few samples (<3.2%). Detection of *Ent. gergoviae* (*P* = 6.3 × 10^−9^) and *C. freundii* (*P*, 0.005) was significantly less common in gecko droppings in offices than from other places. On the other hand, isolation of *E. coli* (*P* = 8.1 × 10^−7^) and *K. pneumoniae* (*P* = 3.4 × 10^−4^) was more common from gecko droppings collected from animal houses. *Salmonella salamae* was detected in samples collected from human houses, while *S. houtenae* was not detected in any of the samples collected from houses, hostels, and guest houses. Detection of *S. indica* was also significantly (*P* = 0.032) lower in samples collected from houses than those from offices and IFS and HGM units. Some of the bacteria including *C. amalonaticus, E. fergusonii, Serratia fonticola*, and *Pragia fontium* were detected in samples collected from households, while *Serratia marcescens* and *Xenorhabdus luminescens* were present in samples collected from offices. *Leclercia adecarboxylata* was present only in samples collected from rabbit houses, while *Ent. aerogenes* and *Hafnea alvei* were present in samples of hostel rooms only.

Isolation of bacteria of different groups varied significantly among gecko faecal dropping samples collected from different places except edwardsiellae which were isolated from samples at all different sources. At least one *Enterobacteriaceae* member was detected in 44 samples from offices and in 70 of the 88 samples collected from different houses. Additionally, one or more members of *Enterobacteriaceae *were isolated from all samples of gecko droppings collected from IFS and HGM units.

### 3.2. Antimicrobial Drug Resistance in Bacteria Isolated from Gecko Droppings

Out of 326 bacterial isolates from gecko faecal droppings, 223 were tested for antimicrobial drug resistance against eight drugs including ampicillin, cefotaxime, ciprofloxacin, chloramphenicol, cotrimoxazole, gentamicin, nitrofurantoin, and tetracycline. Those strains resistant to three or more drugs were classified as multidrug-resistant (MDR) strains. Only 27 (12.1%) isolates were MDR type. Among all major groups of bacteria isolated from gecko droppings, MDR strains were detected (Table 2). However, none of 41 salmonellae (*S. indica* 30, *S. salamae* 7, *S. houtenae* 4) nor 5 edwardsiellae (*Ed. tarda* 4, *Ed. hoshiniae* 1) strains demonstrated MDR. Of the total 223 strains tested, 73 were not resistant to any of the 8 drugs, while 72, 51, 20, and 4 strains were resistant to one, two, three, and four drugs, respectively. Only one isolate each was resistant to 5 (*E. coli*, from rabbitry), 6 (*E*. *coli*, from piggery) and 7 (*E. coli*, from rabbitry) drugs. MDR strains were significantly more common among *Escherichia* spp. (*P* = 1.9 × 10^−5^) and in isolates from IFS units (17 of 27 MDR). Among 51 tested isolates of bacteria collected from IFS, 17 were MDR type; the proportion was significantly (*P* = 3.58 × 10^−23^) higher than that of isolates from samples collected from offices (3 of 51), households (6 of 68), and HGM (1 of 53).

Antimicrobial drug resistance assay results revealed (Table 2) that most of the bacterial isolates from gecko droppings were sensitive to gentamicin (99.1%), ciprofloxacin (98.7%), tetracycline (95.1%), cefotaxime (91.9%), chloramphenicol (91%, cotrimoxazole), and nitrofurantoin (69.5%). However, more than 50% of isolates were resistant to ampicillin. Resistance of different bacterial strains varied significantly (*P* = 2.6 × 10^−4^ to 7.6 × 10^−9^) for all the antimicrobials except for gentamicin, ciprofloxacin, and tetracycline (Table 2).

### 3.3. Antimicrobial Herbal Drug Resistance in Bacteria Isolated from Gecko Droppings

In contrast to antimicrobial drugs, herbal antimicrobials were effective to inhibit growth of only a few bacteria isolated from gecko droppings. Even the most effective herbal drug (Table 3), *Ageratum conyzoides* extract, was less effective than any of the commercially available antimicrobials used in the study (ampicillin). Among antimicrobial herbal drugs, ethanolic extract of *A. conyzoides* was effective on the largest number (27.8%) of strains followed by ethanolic extract of *Zanthoxylum rhetsa *(13.9%), eucalyptus oil (5.4%), patchouli oil (5.4%), lemongrass oil (3.6%), sandalwood oil (3.1%), and *Artemisia vulgaris* essential oil (3.1%). Resistance to herbal drugs significantly (*P* = 0.029 to 4.7 × 10^−18^) varied among strains of different bacteria (Table 3).

Resistance of bacterial isolates to ampicillin (*P* = 0.61), ciprofloxacin (*P* = 0.26), *A. vulgaris* (*P* = 0.62), lemongrass oil (*P* = 0.10), sandalwood oil (*P* = 0.10), and gentamicin (*P* = 0.08) was little affected by the source of sample. However, for other antimicrobials, the source of the sample affected significantly (*P* = 0.024 to 5.6 × 10^−5^) the outcome (Table 4). Though there was no significant effect of source of sample on resistance of bacterial isolates to ampicillin, effect of ampicillin greatly varied *P* = 1.7 × 10^−10^) with the type of bacteria. All strains of *Klebsiella *and* Raoultella* were resistant, and all strains of *Edwardsiella* were sensitive to ampicillin (Table 5). A similar difference was also evident among strains of different bacteria for effect of nitrofurantoin (>65% of klebsiellae, >38% of citrobacteria, and about 25% of *Escherichia* strains were resistant) and cotrimoxazole (>37% of *E. coli* strains were resistant, while <12% strains of other bacteria had resistance). However, efficacy of ciprofloxacin (*P* = 0.62), gentamicin (*P* = 0.87), and *A. conyzoides* (*P* = 0.12) was barely affected by the species or genus of bacteria (Table 5).

## 4. Discussion

It is widely accepted that potentially enteropathogenic and zoonotically important bacteria may be present in intestine of geckos (common house lizards), and thus the geckos have been seen as potential threat in spread of enteric diseases [1–5]. This study was conducted to determine if enteric bacteria are actually present in faecal droppings of geckos, because not all intestinal bacteria are excreted in faeces of geckos.

In the study, bacteria belonging to 34 species of 17 genera were detected in faecal dropping of geckos. Many of the bacteria isolated from faecal droppings of geckos have been reported earlier in intestinal contents including nontyphoidal salmonellae [1–5]; *Citrobacter freundii*, *Enterobacter cloacae* [2, 5], *Edwardsiella tarda, Enterobacter *species, *Serratia marcescens*, *Proteus* spp., *Klebsiella pneumonia*, and *Escherichia coli* [5]. However, many of bacteria reported earlier including several *Salmonella* serovars of *S. enterica* ssp. *enterica* [1, 3], *C. intermedius, Erwinia herbicola* [2, 5], and *Shigella sonnei* [5] could not be isolated possibly due to differences in time and geography of sampling. Instead, several *Salmonella* (*S. indica, S. slamae, and S. houtenae*), *Klebsiella oxytoca*, *Raoultella terrigena*, *Edwardsiella hoshiniae*, *Listonella damsele*, *Leclercia adecarboxylata*, and *Lemionorella ghrimontii* are reported here for the first time from gecko faecal droppings. The isolation of bacteria of 34 species from gecko droppings provides a glimpse in to diversity of faecal microbiota of geckos. The bacterial population in gecko droppings may vary significantly under different environments as droppings collected from geckos living at different places had significant difference in bacterial population of droppings.

Drug resistance has been reported to be of great concern in bacteria isolated from geckos [2, 4, 5], and in the present study 12.1% strains had resistance to three or more drugs. Low rates of drug resistance in bacteria from droppings of geckos observed in the present study might be due to lesser loads of antibiotics in environment. This view is further supported by the fact that >33% bacteria isolated from droppings of geckos residing in IFS units were MDR type. In IFS, the use of antibiotics is common [9]; therefore, isolation of MDR strains from droppings of geckos living in IFS units appears to be non surprising. Thus it can be concluded that it is not the gecko but the environment which prompts the presence of MDR strains. Further, well-designed studies need to be conducted to determine whether or not geckos concentrate and propagate MDR strains or not. Besides environment, inherent drug resistance of certain bacteria might have also contributed to persistence of drug resistant microbes in gecko faeces. All klebsiellae were resistant to ampicillin which is an inherent quality of most the *Klebsiella *and *Raoultella* strains [10].

Resistance to herbal antimicrobials was remarkably high among bacterial strains of gecko origin, and none of the herbal preparations were able to inhibit growth of >27.8% isolates. Herbal drugs are often reported to be important alternatives for MDR strains, and it is shown that bacteria can not develop resistance to herbal medicines [11–13]; however, our observations contradict with these findings. The reason for the contrasting observations might be due to the selection of strains for testing against herbal drugs in earlier studies or might be due to the changing environment. Further, earlier opinions [11–13] might be based on the studies conducted on a few selected bacterial strains, while in our study all isolates of bacteria from gecko droppings were tested, which might have made us to record resistance for herbal preparations in microbes. Moreover, Nagaland is a rich source of herbs in India, and this presence/abundance of herbs in environment may result in the selection of herbal drug resistant strains similar to antimicrobial drugs [11–13]. In the present study, there was a significant difference in sensitivity of different bacteria to herbal drugs indicating that herbal drugs also kill bacteria selectively and are not broad spectra. Similar observations have also been made earlier and enteric bacteria are often reported more resistant than other bacteria to herbal drugs [7, 8, 14–16].

This study concluded that enteric bacteria in gecko faecal droppings and their drug resistance pattern vary significantly depending on geckos' surroundings. Bacteria isolated were usually sensitive to most of the commercially available antimicrobials but resistant to most of the herbal drugs claimed to have good antimicrobial activity.

## Figures and Tables

**Table 1:** Bacterial isolates from faecal droppings of geckos collected from different sources.

Bacterial isolates	Number of gecko samples from different sources positive for bacteria (number of samples tested)					Chi-test statistics	Total number of isolates of bacteria
	Offices *n* = 60	HHH *n* = 88	IFS *n* = 18	HGM *n* = 28	Total positive (%) *N* = 194		
*Citrobacter diversus *	2	1	1	1	5 (2.6%)	0.657	9
*C. freundii *	6	12	4	11	33 (17.0%)	0.005	66
*C. amalonaticus *	0	6	0	0	6 (3.1%)	0.059	11
*Edwardsiella tarda *	0	1	1	1	3 (1.5%)	0.296	4
*Ed. hoshiniae *	1	0	0	0	1 (0.5%)	0.523	1
*Enterobacter cloacae *	1	0	2	0	3 (1.5%)	0.005	3
*Ent. aerogenese *	0	0	0	1	1 (0.5%)	0.114	1
*Ent. agglomerans *	4	1	2	4	11 (5.7%)	0.039	13
*Ent. amnigenus *BG-I	3	0	1	2	6 (3.1%)	0.143	6
*Ent. amnigenus *BG-II	2	0	0	0	2 (1.0)	0.211	3
*Ent. cancerogenus *	0	0	1	0	1 (0.5%)	0.020	1
*Ent. gregoviae *	0	1	2	9	12 (6.2%)	6.3 × 10^−9^	18
*Ent. sakazaki *	0	1	0	0	1 (0.5%)	0.750	1
*Erwinia ananas *	0	2	0	0	2 (1.0%)	0.487	2
*Escherichia blattae *	0	2	3	0	5 (2.6%)	0.001	6
*E. coli *	4	17	12	6	39 (20.1%)	8.1 × 10^−7^	56
*E. fergusonii *	0	3	0	0	3 (1.5%)	0.299	3
*Hafnea alvei *	0	0	0	1	1 (0.5%)	0.114	1
*Klebsiella oxytoca *	0	1	0	1	2 (1.0%)	0.459	2
*K. pneumoniae *	5	7	7	8	27 (13.9%)	3.4 × 10^−4^	41
*Leclercia adecarboxylata *	0	0	1	0	1 (0.5%)	0.020	1
*Leminorella ghirmonti *	1	1	0	0	2 (1.0%)	0.867	2
*Listonella damsele *	1	0	1	0	2 (1.0%)	0.168	3
*Pragia fontium *	0	6	0	0	6 (3.1%)	0.059	6
*Proteus penneri *	1	1	0	0	2 (1.0%)	0.867	2
*Pseudomonas aeruginosa *	0	1	2	0	3 (1.5%)	0.006	3
*Raoultella terrigena *	1	2	0	0	3 (1.5%)	0.793	4
*Salmonella indica *	6	1	3	2	12 (6.2%)	0.032	36
*Salmonella salamae *	0	2	0	0	2 (1.0%)	0.487	7
*Salmonella houtenae *	2	0	1	0	3 (1.5%)	0.173	4
*Serratia entamophila *	2	0	0	0	2 (1.0%)	0.523	3
*Serratia fonticola *	0	1	0	0	1 (0.5%)	0.750	4
*Serratia marcescens *	1	0	0	0	1 (0.5%)	0.523	2
*Xenorhabdus luminescens *	1	0	0	0	1 (0.5%)	0.523	1

Total	44	70	18	28	160	0.003	326

HHH: human households; IFS: integrated farm units; HGM: hostel, guest house, and mess/canteen; BG: biogroup; NT: not tested.

Note: Chi-square test was done to test the null hypothesis that sources from which gecko droppings were sampled had no effect on isolation rate of the bacteria.

**Table 2:** Antimicrobial drug resistance of bacteria isolated from faecal droppings of geckos.

Bacteria	Total isolates tested	Number of isolates resistant to antimicrobial discs of								MDR strains
		A	CTX	C	Cf	Co	G	Nf	T	
*Citrobacter diversus *	3	1	0	0	0	0	0	3	0	0
*C. freundii *	43	23	2	2	1	4	1	15	0	3
*C. amalonaticus *	3	0	0	0	0	1	0	1	0	0
*Edwardsiella tarda *	4	0		0	0	0	0	1	0	0
*Ed. hoshiniae *	1	0	0	0	0	0	0	0	0	0
*Enterobacter cloacae *	3	1	1	0	0	0	0	0	0	0
*Ent. agglomerans *	2	1	0	0	0	0	0	0	0	0
*Ent. amnigenus *BG-I	5	3	0	0	0	0	0	0	0	0
*Ent. amnigenus*BG-II	3	2	1	0	0	2	0	0	0	1
*Ent. cancerogenus *	1	1	1	1	0	0	0	1	0	1
*Ent. gregoviae *	5	4	0	1	0	0	0	3	0	1
*Ent. sakazaki *	1	0	0	0	0	0	0	0	0	0
*Erwinia ananas *	1	0	0	0	0	0	0	0	0	0
*Escherichia blattae *	6	3	1	3	0	5	1	2	0	3
*E. coli *	44	13	7	6	2	14	0	10	8	8
*E. fergusonii *	1	0	0	0	0	0	0	0	0	0
*Hafnea alvei *	1	0	0	0	0	0	0	0	0	0
*Klebsiella oxytoca *	2	2	0	0	0	0	0	1	0	0
*K. pneumoniae *	35	35	2	3	0	4	0	23	1	5
*Leminorella ghrimontii *	1	0	0	0	0	0	0	0	0	0
*Listonella damsele *	3	2	3	0	0	2	0	0	0	2
*Pragia fontium *	4	3	0	0	0	0	0	1	0	0
*Proteus penneri *	2	0	0	1	0	1	0	1	1	1
*Pseudomonas aeruginosa *	3	2	0	3	0	0	0	3	0	2
*Raoultella terrigena *	3	3	0		0	0	0	0	0	0
*Salmonella indica *	30	4	0	0	0	1	0	2	0	0
*Salmonella salamae *	7	7	0	0	0	0	0	0	0	0
*Salmonella houtenae *	4	1	0	0	0	0	0	0	1	0
*Serratia fonticola *	1	0		0	0	0	0	0	0	0
*Serratia marcescens *	1	1	0	0	0	0	0	1	0	0

Total	223	112	18	20	3	34	2	68	11	27

% resistant strains		50.2	8.1	9.0	1.3	15.2	0.9	30.5	4.9	

Chi-test values		7.6 × 10^−9^	2.6 × 10^−4^	5.6 × 10^−5^	0.999	5.6 × 10^−4^	0.911	6.5 × 10^−5^	0.172	9.2 × 10^−3^

A: ampicillin 10 *µ*g; CTX: cefotaxime 30 *µ*g; C: chloramphenicol 30 *µ*g; Cf: ciprofloxacin 5 *µ*g; Co: cotrimoxazole 25 *µ*g; G: gentamicin 10 *µ*g; Nf: nitrofurantoin 300 *µ*g; T: tetracycline 30 *µ*g.

Note: Chi-square test was done to test the null hypothesis that type of bacteria had no effect on resistance to antimicrobial drug.

**Table 3:** Antimicrobial herbal-drug resistance of bacteria isolated from faecal droppings of geckos.

Bacteria	Isolates tested	Number of isolates resistant to						
		AC	AV	EG	LGO	PO	SWO	ZR
*Citrobacter diversus *	3	1	3	2	3	2	3	1
*C. amalonaticus *	3	2	2	3	3	3	3	3
*C. freundii *	43	27	42	42	43	43	42	33
*Edwardsiella tarda *	4	3	4	4	3	4	4	4
*Ed. hoshiniae *	1	1	1	1	1	1	1	1
*Enterobacter cloacae *	3	1	3	2	3	3	3	3
*Ent. agglomerans *	2	2	2	2	2	2	2	2
*Ent. amnigenus *BG-I	5	5	5	5	5	5	5	5
*Ent. amnigenus *BG-II	3	2	2	0	1	1	2	1
*Ent. cancerogenus *	1	1	1	1	1	1	1	1
*Ent. gregoviae *	5	4	5	5	5	4	5	3
*Ent. sakazaki *	1	1	1	1	1	1	1	0
*Erwinia ananas *	1	1	1	1	1	1	1	0
*Escherichia coli *	44	39	44	44	43	42	43	41
*E. blattae *	6	4	6	6	6	6	6	6
*E. fergusonii *	1	1	1	1	1	1	1	1
*Hafnea alvei *	1	0	1	1	1	1	1	0
*Klebsiella oxytoca *	2	0	2	2	2	2	2	2
*K. pneumoniae *	35	27	34	35	35	35	35	32
*Leminorella ghrimontii *	1	0	1	0	1	1	1	1
*Listonella damsele *	3	1	0	0	0	0	0	3
*Pragia fontium *	4	4	4	4	4	4	4	4
*Proteus penneri *	2	2	2	1	2	2	2	2
*Pseudomonas aeruginosa *	3	3	3	3	3	3	3	3
*Raoultella terrigena *	3	1	3	3	3	3	3	1
*Salmonella indica *	30	18	30	29	29	27	29	28
*Salmonella salamae *	7	7	7	7	7	7	7	7
*Salmonella houtenae *	4	2	4	4	4	4	4	4
*Serratia fonticola *	1	0	1	1	1	1	1	0
*Serratia marcescens *	1	1	1	1	1	1	1	0

Total	223	161	216	211	215	211	216	192

% resistant strains		72.2	96.9	94.6	96.4	94.6	96.9	86.1

Chi-test values		0.029	3.3 × 10^−12^	4.7 × 10^−18^	4.9 × 10^−14^	2.9 × 10^−8^	1.5 × 10^−10^	2.8 × 10^−5^

All herbal antimicrobials were used as 0.5 mg/disc; AC: *Ageratum conyzoides* ethanolic extract; AV: *Artemisia vulgaris* essential oil; EG: eucalyptus gum; LGO: lemongrass oil; PO: patchouli essential oil; SWO: sandalwood oil; ZR: *Zanthoxylum rhetsa* ethanolic extract.

Note: Chi-square test was done to test the null hypothesis that type of bacteria had no effect on resistance to antimicrobial drug.

**Table 4:** Effect of source of geckos on antimicrobial drug resistance (% strains) in bacterial isolates from faecal droppings of geckos.

Antimicrobial drug (content in disc)	% Resistant isolates of bacteria isolated from lizards of different sources (number of isolates tested)					Chi-test statistics
	Office (51)	HHH (68)	IFS (51)	HGM (53)	Total (223)	
Ampicillin (10 *µ*g)	47.1	45.6	52.9	56.6	50.2	0.614
Cefotaxime (30 *µ*g)	7.8	2.9	21.6	1.9	8.1	5.2 × 10^−4^
Chloramphenicol (30 *µ*g)	0	10.3	23.5	1.9	9	7.6 × 10^−5^
Ciprofloxacin (5 *µ*g)	0	1.5	3.9	0	1.3	0.264
Cotrimoxazole (25 *µ*g)	9.8	23.5	21.6	3.8	15.2	8.3 × 10^−3^
Gentamicin (10 *µ*g)	0	0	3.9	0	0.9	0.783
Nitrofurantoin (300 *µ*g)	3.9	36.8	41.2	37.7	30.5	5.6 × 10^−5^
Tetracycline (30 *µ*g)	0	2.9	15.7	1.9	4.9	7.6 × 10^−4^
AC (500 *µ*g)	66.7	85.3	82.4	50.9	72.2	1.0 × 10^−4^
AV (500 *µ*g)	94.1	97.1	98	98.1	96.9	0.620
EG (500 *µ*g)	86.3	95.6	96.1	100	94.6	0.016
LGO (500 *µ*g)	92.2	100	98	94.3	96.4	0.100
PO (500 *µ*g)	84.3	100	92.2	100	94.6	4.1 × 10^−4^
SWO (500 *µ*g)	92.2	98.5	96.1	100	96.9	0.104
ZR (500 *µ*g)	90.2	88.2	92.2	73.6	86.1	0.024

AC: *Ageratum conyzoides* ethanolic extract; AV: *Artemisia vulgaris* essential oil; EG: eucalyptus gum; LGO: lemongrass oil; PO: patchouli essential oil; SWO: sandalwood oil; ZR: *Zanthoxylum rhetsa* ethanolic extract; HHH: human house-holds; IFS: integrated farm units; HGM: hostel, guest house and mess/canteen.

Note: Chi-square test was done to test the null hypothesis that source of bacteria had no effect on resistance to antimicrobial drug.

**Table 5:** Variation in antimicrobial drug resistance (% strains) among different groups of bacteria isolated from faecal droppings of geckos.

Antimicrobial drugs used (contents in disc)	% resistant isolates of different groups of bacteria (number of isolates tested)								Chi-test statistics
	*Citrobacter *spp. (49)	*Edwardsiella *spp*. *(5)	*Enterobacter *spp*. *(20)	*Escherichia *spp. (51)	*Klebsiella *spp. (37)	*Salmonella enterica *(41)	Other bacteria (20)	Total (223)	
A (10 *µ*g)	49	0	60	31.4	100	29.3	55	50.2	1.7 × 10^−10^
CTX (30 *µ*g)	4.1	0	15	15.7	5.4	0	15	8.1	0.062
C (30 *µ*g)	4.1	0	10	17.6	8.1	0	20	9	0.033
Cf (5 *µ*g)	2	0	0	3.9	0	0	0	1.3	0.622
Co (25 *µ*g)	10.2	0	10	37.3	10.8	2.4	15	15.2	1.3 × 10^−4^
G (10 *µ*g)	2	0	0	2	0	0	0	0.9	0.870
Nf (300 *µ*g)	38.8	20	20	23.5	64.9	4.9	30	30.5	1.5 × 10^−6^
T (30 *µ*g)	0	0	0	15.7	2.7	2.4	5	4.9	0.008
AC (500 *µ*g)	61.2	80	80	86.3	73	65.9	65	72.2	0.121
AV (500 *µ*g)	95.9	100	95	100	97.3	100	85	96.9	0.046
EG (500 *µ*g)	95.9	100	80	100	100	97.6	75	94.6	4.6 × 10^−5^
LGO (500 *µ*g)	100	80	90	98	100	97.6	85	96.4	0.007
PO (500 *µ*g)	98	100	85	96.1	100	92.7	85	94.6	0.081
SWO (500 *µ*g)	98	100	95	98	100	97.6	85	96.9	0.079
ZR (500 *µ*g)	75.5	100	75	94.1	91.9	95.1	70	86.1	0.005

Other bacteria include isolates of *Erwinia ananas, hafnea alvei, Leclercia adecarboxylata, Leminorella ghirmonti, Listonella damsel, Pragia fontium, Proteus penneri, Pseudomonas aeruginosa, Raoultella terrigena, Serratia fonticola, Serratia marcescens,* and* Xenorhabdus luminescens.*

A: ampicillin; CTX: cefotaxime; C: chloramphenicol; Cf: ciprofloxacin; Co: cotrimoxazole; G: gentamicin; Nf: nitrofurantoin; T: tetracycline; AC: *Ageratum conyzoides* ethanolic extract; AV: *Artemisia vulgaris* essential oil; EG: eucalyptus gum; LGO: lemongrass oil; PO: patchouli essential oil; SWO: sandalwood oil; ZR: *Zanthoxylum rhetsa* ethanolic extract.

Note: Chi-square test was done to test the null hypothesis that type of bacteria had no effect on resistance to antimicrobial drug.
